# Upregulation of Serum miR-629 Predicts Poor Prognosis for Non-Small-Cell Lung Cancer

**DOI:** 10.1155/2021/8819934

**Published:** 2021-03-02

**Authors:** Fayong Liu, Tianshui Li, Ping Hu, Li Dai

**Affiliations:** Department of Respiratory and Critical Care Medicine, Beijing Jishuitan Hospital, Beijing 100000, China

## Abstract

Non-small-cell lung cancer (NSCLC) is one of the most common types of cancer worldwide. Accumulating evidence has suggested that aberrant expression of microRNAs (miRNAs) is involved in the carcinogenesis and progression of NSCLC. The current study is aimed at investigating the clinical significance of serum miR-629 in NSCLC. The expression levels of serum miR-629 in patients with NSCLC, patients with nonmalignant lung diseases, and healthy controls were assessed by real-time quantitative polymerase chain reaction. Our results showed that serum miR-629 levels were significantly upregulated in NSCLC patients compared to the controls. Serum miR-629 exhibited better performance for discriminating NSCLC patients from healthy controls, compared to the traditional biomarkers CYFRA 21-1 and CEA. In addition, a high serum miR-629 level was positively correlated with adverse clinicopathological parameters including lymph node metastasis, differentiation, and clinical stage. Serum miR-629 was dramatically reduced in the NSCLC cases receiving surgical treatment. Moreover, the patients in the high serum miR-629 group suffered poorer overall survival and disease-free survival than those in the low serum miR-629 group. In conclusion, serum miR-629 might serve as a potential prognostic biomarker for NSCLC.

## 1. Introduction

Lung cancer is one the most common cancers and by far the leading cause of cancer-associated mortality in the world. Non-small-cell lung cancer (NSCLC) accounts for approximately 85% of lung cancer [1]. The incidence and mortality rates of NSCLC are still increasing annually in both developed and developing countries [2]. Genetic susceptibility, poor diet, occupational exposures, and cigarette smoking are the major risk factors for NSCLC. Currently, the therapeutic methodologies for NSCLC include surgery, immunotherapy, radiotherapy, and targeted therapy [3]. Although great progress has been made in improving the therapeutic strategies, the prognosis of NSCLC remains poor and the five-year overall survival rate is only 15% [4, 5]. Therefore, developing highly efficient biomarkers for prognosis prediction of NSCLC is critical for improving the clinical outcome of NSCLC.

MicroRNAs (miRNAs) are a class of highly conserved small noncoding RNAs that play a role in posttranscriptional regulation of gene expression [6, 7]. miRNAs have been identified to be involved in diverse biological processes such as cellular differentiation, proliferation, apoptosis, and development processes [8]. In addition, aberrant expression of miRNAs has been observed in different types of human cancer including NSCLC [9]. For instance, overexpression of miR-154 significantly repressed the migration and invasion abilities of NSCLC cells *in vitro*, suggesting that miR-154 may be a potential anticancer therapeutic target for NSCLC [10]. Similarly, miR-101 is reported to act as a tumor-suppressive miRNA in NSCLC [11].

Previous studies have demonstrated that miR-629 was highly expressed in various cancers, such as lung adenocarcinoma, gastric cancer, and pancreatic cancer [12–15]. However, the clinical significance of serum miR-629 in NSCLC is still unclear. Therefore, the aim of the present study was to determine the expression level of serum miR-629 in NSCLC and explore its potential prognostic value.

## 2. Materials and Methods

### 2.1. Subject Recruitment and Sample Collection

This study received ethical approval from the Ethics Committee of Beijing Jishuitan Hospital, and the protocol was in accordance with the Declaration of Helsinki. Written informed consent was obtained from each participant. A total of 166 patients with NSCLC, 70 patients with nonmalignant lung diseases, and 100 healthy volunteers were enrolled in our study. All the NSCLC cases were pathologically confirmed. The detailed information of the participants is shown in Table 1. The patients with nonmalignant lung diseases and healthy controls were matched with NSCLC cases for age and gender. The serum was separated from the blood by centrifugation at 1200 g for 10 min at room temperature. All extracted serum samples were stored at −80°C until further analysis.

### 2.2. RNA Extraction and qRT-PCR

An miRNeasy Serum/Plasma kit (Qiagen, Hilden, Germany) was used to extract the total RNA from serum samples. The RNA quality and concentration were assessed using a NanoDrop™ 1000 Spectrophotometer (Thermo Fisher Scientific, Waltham, MA, USA). The OD_260_/OD_280_ ratios were 1.8-2.2, and the OD_260_/OD_230_ ratios were 2.0-2.2. Complementary DNA was synthesized by reverse transcription reaction using the TaqMan MicroRNA Reverse Transcription Kit (Applied Biosystems, Foster City, CA, USA). Quantitative real-time PCR for the relative quantification of serum miR-629 was performed using a miScript SYBR Green PCR kit (Qiagen) on a 7500 Real-Time PCR System (Applied Biosystems). The PCR conditions were 95°C for 5 min, followed by 40 cycles at 94°C for 15 sec and 55°C for 30 sec. Spiked-in Cel-miR-39 was used as a normalizer for serum miR-629 quantification. The relative level of serum miR-629 was calculated through the 2^–*ΔΔ*Ct^ method.

### 2.3. Enzyme-Linked Immunosorbent Assay

The levels of CYFRA21-1 and carcinoembryonic antigen (CEA) in NSCLC patients and healthy subjects were determined with the CYFRA21-1 ELISA Kit (MyBioSource, San Diego, CA, USA) and Human Carcinoembryonic Antigen ELISA Kit (MyBioSource), respectively.

### 2.4. Statistical Analysis

The differences in the serum miR-629 level between/among different groups were determined by the Mann-Whitney *U*-test or Kruskal-Wallis test. Receiver operating characteristic (ROC) curves and the area under the ROC curve (AUC) were generated to assess the diagnostic accuracy of serum miR-629, CYFRA 21-1, and CEA. The overall survival and disease-free survival were evaluated by Kaplan-Meier analysis and log-rank test. Statistical analysis was performed using GraphPad Prism 7.0 software (GraphPad Prism Software Inc., La Jolla, CA, USA). A *P* value less than 0.05 was considered to be statistically significant.

## 3. Results

### 3.1. Serum miR-629 Levels Were Significantly Upregulated in NSCLC

**Figure 1:**
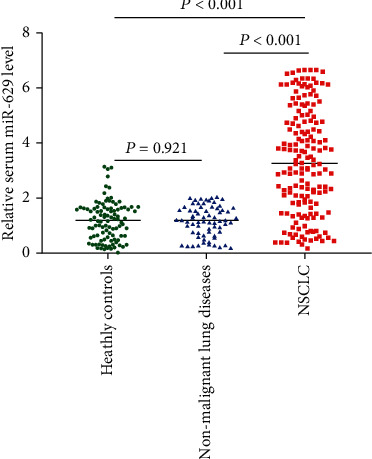
The expression level of serum miR-629 was significantly increased in patients with NSCLC compared to that in nonmalignant lung disease and healthy controls.

The expression level of miR-629 was examined in 166 NSCLC patients, 70 patients with nonmalignant lung diseases, and 100 healthy controls with quantitative RT-PCR. The intra-assay and interassay coefficients of variation (CV) for serum miR-629 were as follows: NSCLC group (intra-assay CV = 2.73%, interassay CV = 3.67%), nonmalignant lung disease group (intra-assay CV = 1.41%, interassay CV = 3.15%), and healthy control group (intra-assay CV = 1.56%, inter-assay CV = 4.22%). Our results showed that the expression level of serum miR-629 was significantly higher in patients with NSCLC than in patients with nonmalignant lung diseases and healthy controls (*P* < 0.001). No significant difference was found between patients with nonmalignant lung diseases and healthy controls (*P* = 0.921) (Figure 1).

### 3.2. The Diagnostic Potential of Serum miR-629 in NSCLC

**Figure 2:**
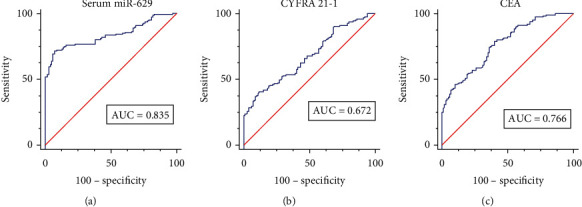
The diagnostic accuracy of serum miR-629 for NSCLC. (a) The diagnostic performance of serum miR-629 for discriminating NSCLC patients from healthy controls. (b) The diagnostic performance of CYFRA 21-1 for discriminating NSCLC patients from healthy controls. (c) The diagnostic performance of CEA for discriminating NSCLC patients from healthy controls.

The diagnostic value of serum miR-629 for NSCLC was performed with ROC curve analysis. The results showed that the AUC value of serum miR-629 for discriminating NSCLC patients from healthy controls was 0.835 (Figure 2(a)), which was higher than that of CYFRA 21-1 (AUC = 0.672) (Figure 2(b)) and CEA (AUC = 0.766) (Figure 2(c)).

### 3.3. The Association between Serum miR-629 Expression and Clinicopathological Features of NSCLC

**Figure 3:**
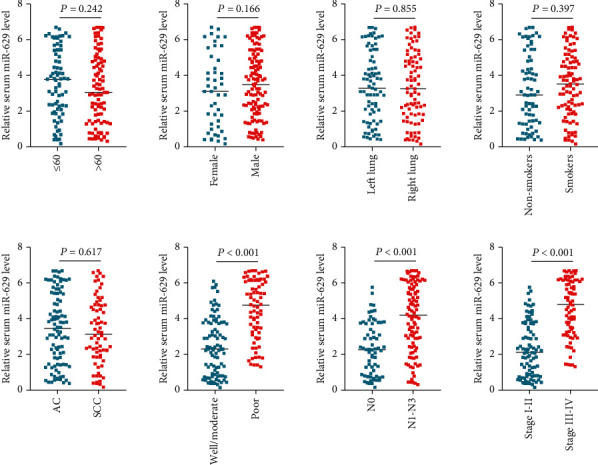
The association between serum miR-629 level and clinicopathological parameters of NSCLC.

As shown in Figure 3, the NSCLC patients with poorly differentiated grade (*P* < 0.001), positive lymph node metastasis (*P* < 0.001), or advanced clinical stages (*P* < 0.001) had significantly higher serum miR-629 levels than those with well/moderate differentiated grade, negative lymph node metastasis, and early clinical stages. In addition, no significant difference was found between serum miR-629 and other analyzed clinicopathological parameters including age (*P* = 0.242), gender (*P* = 0.166), primary location (*P* = 0.855), smoking history (*P* = 0.397), and histological type (*P* = 0.617).

### 3.4. The Association between Serum miR-629 Expression and Therapeutic Response as well as Survival

**Figure 4:**
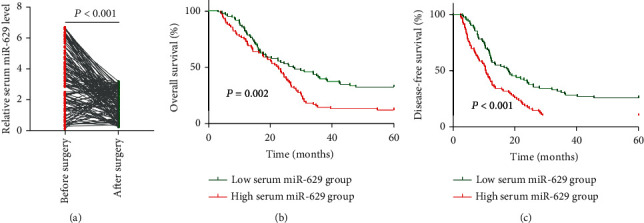
The association between serum miR-629 level and therapeutic responses as well as survival in NSCLC. (a) Serum miR-629 level was significantly increased following surgical treatment. (b, c) The patients in the high serum miR-629 group had worse overall survival and disease-free survival than those in the low serum miR-629 group.

A total of 135 NSCLC patients in this cohort received surgical treatment. We then compared the level of serum miR-629 in pretreated samples and posttreated samples. Our results showed that the level of serum miR-629 was markedly decreased in NSCLC patients following surgical treatment (*P* < 0.001) (Figure 4(a)). According to the median expression level of serum miR-629, the NSCLC patients were divided into a high-expression group (*n* = 83) and a low-expression group (*n* = 83). Kaplan-Meier analysis demonstrated that patients in the high serum miR-629 group had a significantly shorter overall survival than those in the low serum miR-629 group (*P* = 0.002) (Figure 4(b)). In addition, patients with a higher level of serum miR-629 had worse DFS compared with those with a lower level of serum miR-629 (*P* = 0.011) (Figure 4(c)).

## 4. Discussion

In this study, we have shown that serum miR-629 was significantly upregulated in patients with NSCLC. In addition, serum miR-629 well differentiated patients with NSCLC from healthy controls. Moreover, serum miR-629 expression was notably decreased after treatment. NSCLC patients with higher serum miR-629 expression had worse clinical characteristics and prognosis. These findings might suggest that serum miR-629 might serve as a novel potential prognostic biomarker for NSCLC.

Consistent with our findings, a positive correlation was found between the level of miR-629 and tumor invasion in lung adenocarcinoma (LUAD) specimens. In addition, overexpression of miR-629 enhanced the invasive capacity of lung cancer cells, indicating that miR-629 plays a tumor-promoting role in LUAD [12]. Similarly, miR-629-3p was also found to be upregulated in LUAD tissues and cell lines. Enforced expression of miR-629-3p promoted cell proliferation via downregulating the expression of SFTPC [13].

Aberrant expression of miR-629 has also been reported in other types of cancer. For instance, Li et al. demonstrated that the expression of miR-629 was upregulated in gastric cancer. Ectopic expression of miR-629 suppressed gastric cancer cell proliferation and promoted apoptosis by targeting FOXO3 [14] Similarly, Yan et al. showed that miR-629 expression is markedly upregulated in pancreatic cancer tissues. miR-629 overexpression enhanced cell proliferation and metastasis of pancreatic cancer cells *in vitro* and *in vivo* [15]. Shi et al. reported that miR-629 was significantly increased in the tissues and serum samples from pancreatic cancer patients. In addition, serum miR-629 was demonstrated to be a promising biomarker for the diagnosis and prognosis of pancreatic cancer [16]. The expression of miR-629 was also strongly associated with tipifarnib resistance in breast cancer cell lines [17]. Tao et al. reported that miR-629 was significantly upregulated in hepatocellular carcinoma (HCC) tissues compared with adjacent normal tissues. In addition, an *in vitro* assay showed that overexpression of miR-629 promoted the proliferation, migration, and invasiveness of HCC cells, while knockdown of miR-629 led to the opposite effects [18]. In colorectal cancer, miR-629 was found to be significantly upregulated in colorectal cancer tissues and cell lines, and upregulation of miR-629 enhanced cell proliferation and migration as well as suppressed cell apoptosis by directly downregulating CXXC4 [19].

One of the potential limitations of our study was the relatively small sample size. Further studies with larger sample sizes are urgently needed to validate our findings. In addition, as we have discussed above, serum miR-629 might be also upregulated in other types of cancer or other human diseases. Therefore, combining the serum miR-629 and currently known biomarkers for NSCLC as well as clinical parameters might contribute to improving the diagnosis and prognosis prediction of NSCLC. Moreover, the molecular mechanisms accounting for the tumor-promoting role in NSCLC need further investigation.

In conclusion, to the best of our knowledge, this is the first study to report that serum miR-629 is upregulated in NSCLC. An increased level of serum miR-629 is associated with a poor clinical outcome of NSCLC, indicating that serum miR-629 might serve as a promising and novel prognosis biomarker for NSCLC.

## Figures and Tables

**Table 1:** The clinical information of the study cohort.

Parameters	NSCLC	NMLDs	Healthy controls
Age	62.35 ± 8.52	63.17 ± 9.05	61.45 ± 7.56
*Gender*			
Male	119	48	64
Female	47	22	36
*Smoker*			
Yes	93	38	56
No	73	32	44
*Primary location*			
Left lung	79	33	—
Right lung	87	37	—
*Histological type*			
AC	96	—	—
SCC	70	—	—
*Differentiation*			
Well/moderate	92	—	—
Poor	74	—	—
*Lymph node metastasis*			
Negative	66	—	—
Positive	100	—	—
*Stage*			
I-II	88	—	—
III-IV	78	—	—

## Data Availability

The data that support the findings of this study are available from the corresponding author upon reasonable request.
